# Ultrasonographic assessment of inferior vena cava/abdominal aorta diameter index: a new approach of assessing hypovolemic shock class 1

**DOI:** 10.1186/s12245-016-0101-z

**Published:** 2016-02-19

**Authors:** Nik Hisamuddin NA Rahman, Rashidi Ahmad, Meera Mohaideen Kareem, Mohammad Iqhbal Mohammed

**Affiliations:** Department of Emergency Medicine, School of Medical Sciences, USM, Kota Bharu, Malaysia; Unit of Emergency Medicine, UM Medical Center, Kuala Lumpur, Malaysia; Department of Radiology, School of Medical Sciences, USM, Kota Bharu, Malaysia

**Keywords:** Shock, Inferior vena cava, Hypovolemia

## Abstract

**Background:**

We designed this study to expand the usage of ultrasound to detect early occurrence of hypovolemia. We explore the potential use of inferior vena cava (IVC) and abdominal aorta (AA) diameter index (IVC:AA) measured ultrasonographically to detect class 1 hypovolemic shock with blood loss less than 15%.ᅟ

**Methods:**

The aim of this study was to determine the changes in the diameter of inferior vena cava and abdominal aorta in blood donors by using ultrasound, pre and post blood donation. The result of the study would be further explorated to determine the inferior vena cava (IVC) abdominal aorta (Aorta) diameter index (IVC:Aorta). This was a prospective study done in the blood bank of a university hospital. Researcher was trained by a senior radiologist to assess inferior vena cava and abdominal aorta diameter. Fifty-two healthy blood donors were included in the study. Inclusion criteria were same with the blood bank criteria to donate blood. Demographic data and vital signs were taken before the ultrasound measurement done for inferior vena cava and abdominal aorta diameter. Once the volunteers donated their blood of approximately 450 mls; the measurements were repeated using the same methods.

**Results:**

There were differences in IVC, abdominal aorta and inferior vena cava:aorta diameters index pre and post donation. With mathematical analysis, we suggested the number of IVC:Aorta index as 1.14±2SD with SD 0.18 as a cut off value for class 1 hypovolemic shock.

**Conclusion:**

The IVC:Aorta diameter index can be used as a parameter for detecting early phase (Class 1) of hypovolemic shock.

1. Why is this topic important?
  Early detection of hypovolemia in trauma victims is crucial in the early phase of management.
2. What does this study attempt to show?
  It attempts to show that the use of bedside ultrasound can detect early phase of hypovolemia by measuring the diameter of inferior vena cava and abdominal aorta.
3. What are the key findings?
  The IVC:aortic index of 1.14 indicates class 1 hypovolemia.
4. How is patient care impacted?
  The early use of bedside ultrasonography is a very promising *non invasive* and *fast technique* to detect early phase of hypovolemia and hence may *save more lives and reduce morbidity*.

## Background

Trauma is the leading cause of mortality and morbidity in the reproductive age group. The mortality and morbidity are mainly attributed to hypovolemic shock [1]. Hypovolemia results in a reduction of systemic venous return, causing reduction in the stroke volume, which is responsible for the decrease in cardiac output. Commonly the classical clinical features of hypovolemia are absence in the early phase of hemorrhagic shock. Furthermore, the presence of documented hypotension, tachycardia or signs of tissue hypo perfusion are insufficient to confirm the diagnosis of hypovolemia as they are non-specific [2]. Laboratory parameters such as metabolic acidosis, high urea level and haemoconcentration are neither non-sensitive nor specific [3]. Thus, the above parameters should not be relied heavily in clinical approach. The outcome of patients in traumatic shock depends significantly on the early detection of hypovolemia, early fluid resuscitation and definitive corrective measure of the source of bleeding. The early, non-invasive and bedside investigation will enhance the effective management and hence better clinical outcome. It is in this regard we propose the use of the inferior vena cava to abdominal aortic (IVC:AA) diameter index as a new and relevant tool in emergency department (ED) to assess hypovolemia in its early stage. Unfortunately, evidence based on this topic is very scarce. Among the earliest study to assess the IVC:AA index in hypovolemia is carried out by Kosiak et al. [4]. In that study, 52 volunteers aged between 20 and 25 had their IVC and aorta diameter measured before any intervention. They were then rehydrated with 1.5 to 2 l of fluid. The 1.5 to 2 l of fluid loss is equivalent to class II to class III hypovolemia or 30 to 40 % fluid loss. However, the purpose of our study is mainly to develop a new approach in identifying hypovolemic shock at an early phase (class 1 hypovolemic shock) by measuring the ratio between inferior vena cava diameter (IVCD) and abdominal aortic diameter (AAD) using ultrasound machine. We hypothesise that there is a significant change in the ratio of IVCD to the AAD (IVCD:AAD) before and after 450 to 500 ml of blood loss (class 1 hypovolemic shock). The best replication of type I hypovolemia in a controlled environment is among blood donors. We hope the result from this study will enhance the process of reaching accurate diagnosis of early hypovolemic phase and facilitate the initiation of definitive management and treatment in hoping to reduce the mortality and morbidity, either in post-traumatic condition or in any bodily fluid-deprived condition.

### Objectives

The aim of this study is to determine the changes in the diameter of IVCD and AAD in blood donors by using ultrasound, before and after blood donation. The result of the study will be further explorated to determine the IVC:AA diameter ratio (IVC:AA index) before and after blood donation, and subsequently to explore potential use of new method in assessing class 1 hypovolemia. We hypothesise that there is an obvious difference in the IVC:AA diameter index pre and post blood transfusion.

## Methods

**Fig. 1:**
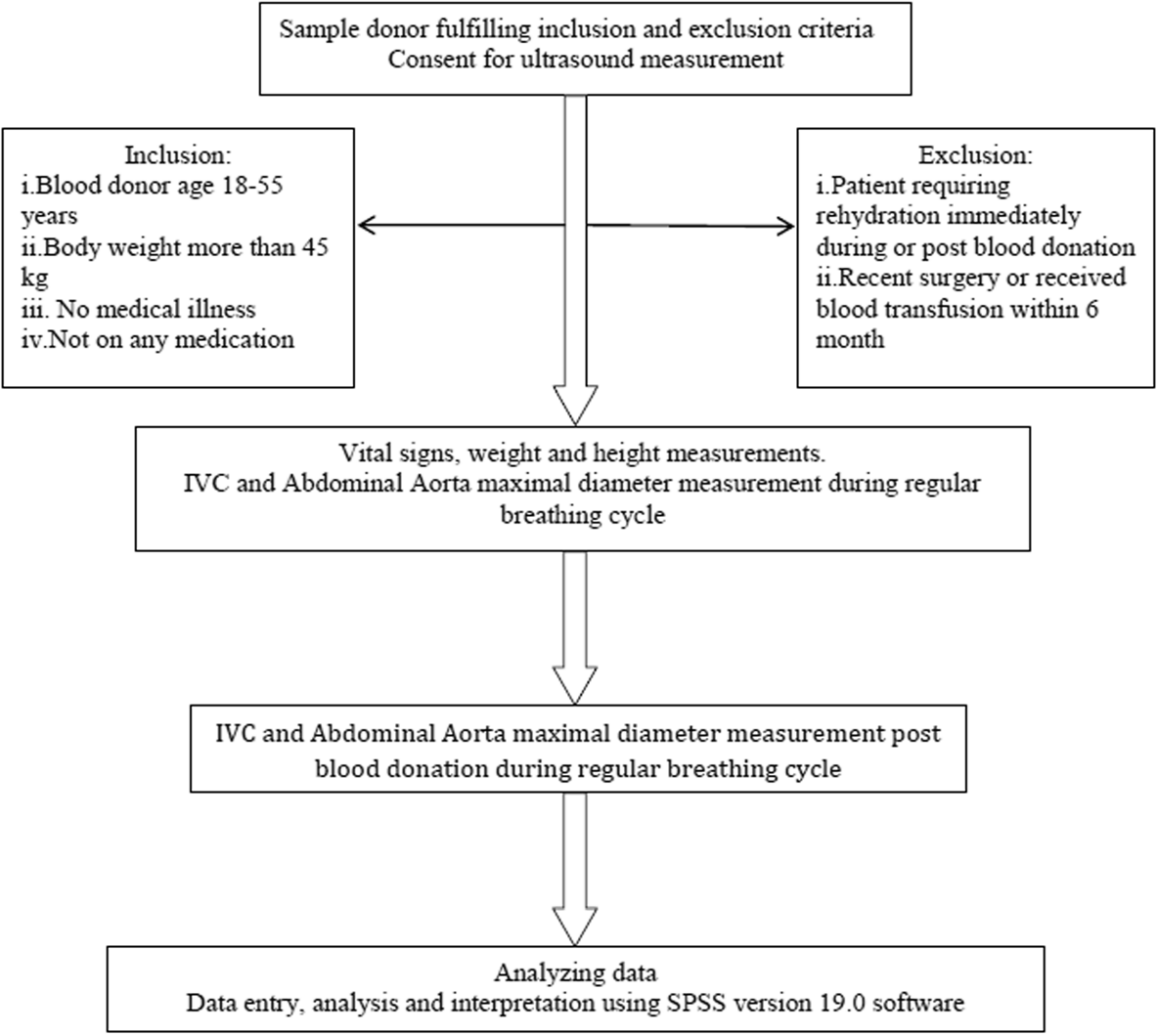
Sample donor fulfilling inclusion and exclusion criteria

This was a cross-sectional, prospective, observational study, conducted on volunteers who attended the blood bank unit of a tertiary teaching university hospital for blood donations over a 3-month period in 2012. The study was approved by the institution board review and hospital ethics committee (USMKK/PPP/JEPeM [223.3. (12)]. Further approval was obtained from the head Department of Hematology for the conduct of the study within the blood bank unit which was under their jurisdiction. Blood donors were identified by the blood bank staff, and all the necessary tests were carried out according to the blood bank protocol. We included subjects that followed the blood donor’s criteria set by the blood bank unit. Blood donor’s criteria include age 18–55 years, weight more than 45 kg, no medical illness, not on any medications, and able to understand and give inform consent. Exclusion criteria included those patient requiring rehydration immediately during or post blood donation, previous surgery or received blood transfusion within 6 months, donor with high risk behaviour such as multiple sex partners or sharing needle and also sex workers (Fig. 1).

As no previous studies were conducted in similar manner, we applied the central limit theorem theory in this study. The ultrasound examination was performed by the researcher who was familiar with ultrasound. The researcher, who was the second year trainee in emergency medicine specialty programme, had obtained necessary testimonial validation by a consultant radiologist. The chosen consultant radiologist, acted as a gold standard sonographer, was a senior faculty member of the radiology department. The researcher underwent intensive 2-week training with the consultant radiologist on the use of ultrasound on human models and further 2 weeks on blood donors in the blood bank unit. The principal of abdominal ultrasound and hands on training, in particular in IVCD and AAD measurement, were carried out on a total of 40 volunteers. The consultant demonstrated the appropriate technique on the first few patients, followed by the researcher himself performing in front of the consultant. In subsequent patients, the researcher will do the initial measurement, followed by the consultant, in order to verify and validate the researchers finding. Objectively, by using the Spearmen’s rank correlation coefficient, there was a positive and significant correlation between the researcher and the radiologist score. The subsequent measurement was done by researcher alone on the real participants.

**Fig. 2:**
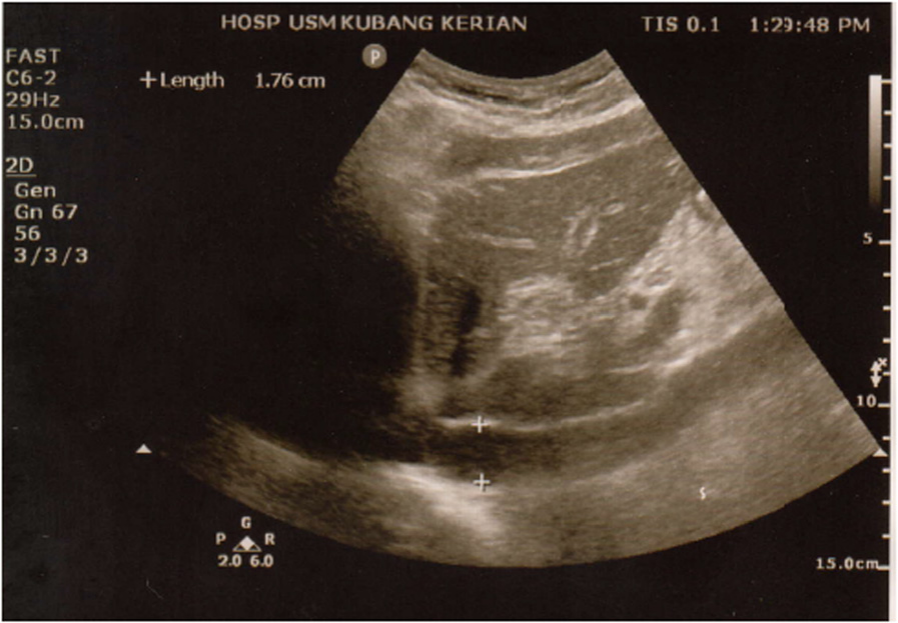
The IVCD was measured during regular breathing cycle

**Fig. 3:**
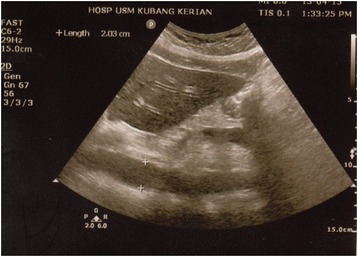
The AAD was measured 10 mm above the celiac trunk

The vital signs were taken by the nurses in charge in the blood bank unit. The height and weight were also recorded. They were subsequently placed on an adjustable couch. The ultrasound measurement was carried out once the donor was in comfortable supine position. The measurement evaluation was done with an ultrasound machine Premium Hand-Carried Colour Doppler Diagnostic Mindray® Model M5 fabricated in China. Curvilinear probe 3.5 to 5 MHz was used with B mode scan. The probe was placed underneath the xiphoid process in a longitudinal position. The IVCD was measured 2 cm from the junction of right atrium inlet, where its anterior and posterior walls were parallel. The IVCD was measured during regular breathing cycle, and the maximum value was recorded (Fig. 2). The AAD was measured 10 mm above the celiac trunk (Fig. 3). Three readings were taken to get the mean value for both diameters. Donors then donated their blood with the standard amount of 450 ml (ml). Similar measurements were repeated after blood donation. The maximum time gap estimate for pre and post donation measurement was 30 min.

Demographic data consisted of gender, age, race, weight, height, shock index (SI) and body mass index (BMI) were recorded. All data were analysed with Statistical Package for Social Sciences software version 18.0 registered to the medical school. The primary variables measurement include the IVCD, AAD and IVCD:AAD ratio index. The observed primary outcome was the difference of IVCD:AAD ratios pre and post blood donation which was analysed by using paired *t* test. The secondary data included the blood pressure and pulse rate pre and post blood donation. Correlation studies were carried out between the BMI and SI and the IVCD, AAD and IVCD:AAD index. Numerical variables are checked for normality and presented as mean and standard deviation. *p* value of less than 0.05 was considered significant statistically.

## Results

Fifty-two donors were included into the study. The general demographic parameters for all the donors are shown in Table 1. The mean IVCD pre donation was 1.75 ± 0.24 cm. Post blood donation mean IVCD was 1.59 ± 0.20 cm (*p* < 0.05). The means AAD pre and post donation were 1.46 ± 0.15 cm and 1.41 ± 0.18 cm, respectively (*p* = 0.035). The mean change of IVCD and AAD pre and post donation were 0.16 ± 0.18 cm (*p* = 0.014) and 0.05 ± 0.12 cm (*p* < 0.05), respectively.

**Table 1:** Weight, height, BMI, systolic BP and heart rate, distribution (mean)

Variables	Mean (SD)	*p* value
Weight (kg)	72.84 (13.80)	Not applicable
Height (m)	1.66 (0.09)	Not applicable
BMI (kg/m^2^)	26.53 (5.70)	Not applicable
Pre donation systolic blood pressure (MmHg)	132.77 ± 11.99	*p* = 0.33
Post donation systolic blood pressure (MmHg	129.10 ± 13.45	
Pre donation heart rate (per minute)	74.29 ± 9.95	*p* = 0.001
Post donation heart rate (per minute)	69.23 ± 9.92	
Pre donation shock index (heart rate/SBP)	0.57 ± 0.08	*p* = 0.17
Post donation shock index (heart rate/SBP)	0.54 ± 0.09	

The IVCD:AAD index was calculated by dividing the mean IVCD to the mean AAD. The mean of IVCD:AAD index pre donation was 1.21 ± 0.20. The post donation index mean was 1.14 ± 0.18. The mean index difference was 0.07 (0.03, 0.11) with *p* = 0.001 (Table 2). We also analysed whether there was any correlation between age and IVCD or AAD by using simple linear regression test. Both revealed no significant correlation between IVCD and AAD pre donation with age (*p* value >0.05). There were also no significant correlation between BMI and Shock Index with IVCD, AAD and IVCD:AAD index for both pre and post blood donation (Tables 3 and 4).

**Table 2:** Paired *t* test for IVC diameter, aortic diameter and IVC:aortic index

	Mean (95 % CI)	t	df	*p* value
Pre IVC—post IVC	−0.16 (0.11, 0.21)	6.6	51	<0.05
Pre aorta—post aorta	−0.05 (0.02, 0.08)	2.9	51	0.035
Pre index—post index	0.07 (0.03, 0.11)	44.1	551	0.01

Paired *t* test for pre and post blood donation

**Table 3:** Linear regression correlating BMI, IVC diameter difference, aortic diameter difference and IVC:aortic index difference

Variable	Coefficient B	SE	*p* value
Intercept BMI and IVC difference	0.197 −0.001	0.119 0.004	0.77
Intercept BMI and aortic difference	0.060 0.000	0.082 0.03	0.90
Intercept BMI and IVC:aortic index difference	26.575 −0.825	0.863 6.605	0.90

**Table 4:** Linear regression correlating shock index (SI), IVC diameter difference, aortic diameter difference and IVC:aortic index difference

Variable	Coefficient B	SE	*p* value
Intercept SI and IVC difference	0.081 0.151	0.158 0.289	0.60
Intercept SI and aortic difference	−0.035 0.158	0.108 0.198	0.43
Intercept SI and IVC:aortic index difference	0.067 0.007	0.112 0.205	0.97

## Discussion

The ability of emergency physician to differentiate hypovolemic shock with the other type of shock will facilitate the initial management and resuscitation without the need to endeavour into much complicated and invasive procedures. Over reliance on classical signs and symptoms of hypovolemia as those summarised by Advance Trauma Life Support (ATLS) will lead to myriads of possibilities and differential diagnosis of the underlying causes [5]. Likewise, the biochemical markers, even some are useful, are of limited use in ED, essentially due to time factor [6]. The ATLS guideline for hypovolemic shock classification which has the stepwise vital sign categorization has been used widely in order to facilitate clinician evaluating hydration or volume status [7]. However, this classification is not fully supported by robust evidence based [8, 9].

The use of ultrasound in trauma is advocated primarily in discovering the presence of free fluid in the dependant area of intraperitoneal cavity, pericardial sac, thoracic cavity and the possibility of tissue damage in the liver and spleen [10]. The specificity and sensitivity of ultrasound in detecting intraperitoneal free fluid is 86 and 99 %, respectively [11, 12]. However, the amount of fluid itself is not predictable through ultrasound [13]. Ultrasonographically measured IVC diameter was recognised as a non-invasive way to assess fluid status in critical care and acute setting. It was pioneered by nephrologists and then expanded further by cardiologist and emergency physician. Recently, the IVC diameter (IVCD) measurement is used to assess intravascular fluid status in patients who are stable haemodynamically but potentially may dwell into profound shock [14]. The change of the IVCD is very well explained by the nature of the structural wall of the vessel itself. It is a highly compliant vessel with good sensitivity to changes in intravascular volume [15]. Studies had shown repeatedly that the IVCD changes before there are any apparent detectable changes in vital signs [16, 17]. This has rendered the use of IVCD measured ultrasonographically becoming more popular among the fraternity of critical care and acute medicine, including in emergency department.

To our knowledge, no study was done before with regard to the IVCD:AAD index measurement in the early of hypovolemia (class I shock). Our study has further confirmed and enhances Kosiak’s study and its applicability in clinical setting. In our study, the focus is on the ratio or index of inferior vena cava to aorta diameter (IVCD:AAD). We believe that in order to obtain a valid result of the parameter, the emphasis should be on the standardisation of measurement method of IVCD:AAD, rather than relying much on respiratory cycle. Further, it is also a question of feasibility in ED setting to measure IVCD in certain phase of respiration. Physiologically, the AAD does change with cardiac cycle and with fluid loss [18]. The changes are the result of interaction between intravascular fluid volume and the compliance of the wall. To our knowledge, there are very limited or absence of studies done on assessing the aorta diameter, in particular relating to the IVCD:AAD index level in hypovolemic state [19]. Agreeably, the range of size obtained in this study might be slightly lower than those found from other studies [20]. This might be attributed to smaller physical size of Asian population. Similar observation of aortic diameter variance was reported among black population in the USA [21]. With mathematical calculation, we propose IVCD:AAD index cut-off point for hypovolemic shock class I as 1.14. Any index measurement below the value given should be considered as fluid deprived and in the early phase of hypovolemic shock (class I shock). Our study has proven the use of the index to diagnose hypovolemic shock at the very early stage. We hope the use of sonography among critically ill patients can be an adjunct apparatus in assisting the physician to diagnose and manage patients in a more efficient way. The dramatic growth of ultrasound use in ED has led to the development of various ultrasonography techniques, protocols and algorithms in order to expedite diagnosis with high accuracy and reliability [22, 23]. Some even view ultrasound as a tool comparable to stethoscope in daily clinical practice. The routine use of ultrasound in trauma through focused assessment with sonography in trauma (FAST), which is incorporated as an adjunct to primary survey and resuscitation in ATLS manual, has made the presence of the machine in ED as a must [24, 25]. We suggest that the FAST should also include the assessment of IVCD and AAD as part of the assessment of hypovolemia post trauma. We also suggest the development of software in the ultrasonography machine that automatically calculates the IVCD:AAD index. The use of the sonography in the assessment of fluid loss is ideally carried out as early as possible such as during the prehospital care and at the triage counter in the ED. This technique can be easily taught among the paramedics and the nurses with minimal difficulty [26].

Few limitations arise from our study. This study was conducted in relatively healthy donors, who have no other confounding factors and co-existing clinical issues, which is not the case in real situation. The above result was not tested in intubated or ventilated patient. We expect the result might vary in view of changes in intra-thoracic pressure and other physiological changes. In assessing intra-abdominal structure with ultrasound, the main limitation is the bowel gas and variability in anatomical position. Despite of the limitations, this study can be a preliminary step in providing new insight of the utility of IVCD:AAD index in mild hypovolemic state and with the ease of use and as a non-invasive method, we strongly recommend that this method of assessing the early fluid loss in any condition can be further explored in a bigger scale research especially in the real ED setting.

## Conclusions

The ultrasonography use in trauma patients has become increasingly important as a bedside assessment tool which is non-invasive and quick. The use of IVC:AA diameter index in detecting the early phase of hypovolemia is a promising technique to detect mild hypovolemia and should be studied in bigger research capacity in the near future.
